# Contextual Fear Conditioning and Fear Generalization in Individuals With Panic Attacks

**DOI:** 10.3389/fnbeh.2019.00152

**Published:** 2019-07-17

**Authors:** Dorothea Neueder, Marta Andreatta, Paul Pauli

**Affiliations:** ^1^Department of Psychology (Biological Psychology, Clinical Psychology, and Psychotherapy), University of Würzburg, Würzburg, Germany; ^2^Center for Mental Health, Medical Faculty, University of Würzburg, Würzburg, Germany

**Keywords:** contextual fear conditioning, anxiety generalization, startle response, panic disorder, virtual reality

## Abstract

Context conditioning is characterized by unpredictable threat and its generalization may constitute risk factors for panic disorder (PD). Therefore, we examined differences between individuals with panic attacks (PA; *N* = 21) and healthy controls (HC, *N* = 22) in contextual learning and context generalization using a virtual reality (VR) paradigm. Successful context conditioning was indicated in both groups by higher arousal, anxiety and contingency ratings, and increased startle responses and skin conductance levels (SCLs) in an anxiety context (CTX+) where an aversive unconditioned stimulus (US) occurred unpredictably vs. a safety context (CTX−). PA compared to HC exhibited increased differential responding to CTX+ vs. CTX− and overgeneralization of contextual anxiety on an evaluative verbal level, but not on a physiological level. We conclude that increased contextual conditioning and contextual generalization may constitute risk factors for PD or agoraphobia contributing to the characteristic avoidance of anxiety contexts and withdrawal to safety contexts and that evaluative cognitive process may play a major role.

## Introduction

Panic disorder (PD) is one of the most disabling of anxiety disorders affecting about 7.8 million individuals in Europe (Wittchen et al., 2011). Acute panic attacks (PA) of intense fear and anticipatory anxiety towards forthcoming attacks are constitutive symptoms of PD (DSM-5, American Psychiatric Association, 2013). Oversensitivity to unpredictable aversive events, but not towards predictable threat, is assumed to be a risk factor for PD (Grillon et al., 1994, 2009; Grillon, 2008; Nelson et al., 2013; Gorka et al., 2017). Consequently, PD patients are assumed to respond with sustained fear in the context in which unpredictable threat was experienced and therefore is expected again, and the concomitant hypervigilance towards potential threat is presumed to facilitate the reoccurrence of additional PA (Başoğlu et al., 1994).

Overgeneralization of conditioned fear responses to stimuli that resemble the original threat-eliciting stimulus (generalization stimuli, GSs) but have never been paired with aversive events is discussed as another risk factor for PD (Lissek, 2012; Dymond et al., 2015). Such overgeneralization of fear in PD was found for discrete stimuli predicting threat but was not examined for contexts associated with unpredictable threat so far, although the latter seems highly relevant for PD.

Following the seminal studies of Baas et al. (2004, 2008), we developed a differential context conditioning paradigm realized with virtual reality (VR), which allows participants to immerse in various ecologically valid computer-based virtual environments (Tröger et al., 2012; Glotzbach-Schoon et al., 2013a). Here, a specific context (anxiety context, CTX+) becomes associated with unpredictable threat and later induces sustained anxious apprehension, while another context (safety context, CTX−) never becomes associated with threat and therefore implies safety. Using this VR paradigm, we successfully demonstrated context conditioning in healthy individuals, thus they rated CTX+ vs. CTX− more anxiogenic, arousing, negatively valenced, and more strongly associated with the unconditioned stimuli (US), and they avoided entering CTX+ (Glotzbach et al., 2012; Glotzbach-Schoon et al., 2013b,c). In addition, we observed startle potentiation and higher skin conductance level (SCL) in CTX+ vs. CTX− (Glotzbach-Schoon et al., 2013b,c; Andreatta et al., 2015a, 2017; Genheimer et al., 2017) as well as stronger amygdala activation (Andreatta et al., 2015b). Importantly, we also found that trait anxiety, an assumed risk factor for anxiety disorders, modulates context conditioning (Glotzbach-Schoon et al., 2013c).

Recently, we advanced our VR paradigm to study the generalization of contextual anxiety. By implementing a generalization context (G-CTX) that shares physical properties of CTX+ and CTX− equally, we were able to reveal generalization of verbal anxiety responses to G-CTX in healthy individuals and we found first indications that trait anxiety modulates generalization as well (Andreatta et al., 2015a).

The current study is that first examining individuals characterized by PA in comparison to healthy controls (HC) regarding context conditioning and generalization of conditioned anxiety. First, we hypothesized that individuals with PA show heightened context conditioning as assessed by verbal and physiological responses during the acquisition phase. Second, we expected that these individuals during the generalization test show persevering anxiety, i.e., greater CTX+ vs. CTX− differences, and overgeneralization of anxiety responses, i.e., greater G-CTX vs. CTX− differences.

## Materials and Methods

### Participants

The final sample consisted of 21 individuals with current PA and 22 HC. Participants were recruited *via* advertisement on public websites and newspapers and were first screened by phone regarding experienced PA. Participants with PA had to report at least one PA during the last 2 weeks and HC had to be free of PA. Additional exclusion criteria were Beck Depression Inventory (BDI, Hautzinger et al., 2006) scores above 16 or above 9 for PA and HC, respectively. Because of the high comorbidity between PD and major depression (Roy-Byrne et al., 2000) it is virtually impossible to recruit individuals with PA without any symptoms of depression. However, as we did not want to examine participants with dominant depressive symptoms, we excluded PA participants who fulfilled clinically relevant criteria of depression as indicated by a BDI above 16. For HC, we considered a BDI score of above 9 as indicating sub-clinical symptoms of depression.

Exclusion criteria for all participants were current use of psychoactive drugs or psychotherapy, severe neurological diseases or pregnancy. Ten participants had to be excluded due to high BDI scores (*n* = 3), motion sickness (*n* = 2), or negligible startle responses (*n* = 5; mean startle amplitude ≤5 mV). All participants gave informed consent approved by the Ethics Committee of the Medical Faculty of the University of Würzburg and were compensated for their participation with 30€.

### Material and Apparatus

Unconditioned stimuli (US) were mildly painful electric stimuli delivered with a frequency of 50 Hz and 200 ms duration by a constant current stimulator (Digitimer DS7A, Digitimer Limited; 400 V, max 9.99 mA) to the dominant forearm *via* two gold-plated stainless-steel surface electrodes of 9 mm diameter and 30 mm spacing. Intensity was individually adjusted in two ascending and descending series (Andreatta et al., 2010) and then increased by 30% to avoid habituation.

Contextual stimuli (CTX) were virtual rooms designed with the Source Engine (Valve Corporation) with identical spatial surface but different layout (for details see Andreatta et al., 2015a) and presented *via* a Z800 3D Visor head-mounted display (HMD, eMagin) controlled by the software CyberSession (VT+ GmbH, Germany).

Acoustic startle probes were 103 dB bursts of white noise presented for 50 ms binaurally *via* headphones.

#### Ratings

Participants rated the valence, arousal, anxiety and contingency they had experienced in the virtual rooms on a visual analog scale (VAS) ranging from 0 to 100 at predefined time points during the experiment (see “*Procedure*” section). More detailed, according for valence 0 depicted negative valuation, 100 positive valuation of the respective room. In arousal 0 stood for no arousal up 100 for very high arousal during the visit of the room. In anxiety 0 mean no anxiety at all, 100 very high anxiety. For contingency, VAS ranged from 0 (no US expectation) to 100 (US surely expected).

Participants were labeled “contingency aware” if the CTX+ minus CTX− difference in contingency ratings was ≥70.

#### Questionnaires

All participants completed the four sub-questionnaires of the Comprehensive Panic Profile (CPP, Clum et al., 1995), a composite measure that reliably evaluates panic symptoms and outcomes (Clum et al., 1990). The Panic Frequency Scale (PFS), Panic Attack Symptoms Questionnaire (PASQ), Panic Attack Cognitions Questionnaire (PACQ), and Avoidance Questionnaire (AQ) were used to quantify the number of PA, the severity of symptoms and the degree of preoccupation with typical cognitions during a PA and the level of avoidance of panic-related places and situations, respectively (Clum et al., 1990, 1995). Furthermore, participants completed the Anxiety-Sensitivity Index (ASI, Peterson and Reiss, 1992; German Version: Alpers and Pauli, 2001), the State-Trait Anxiety Inventory (STAI, Laux et al., 1981), the Positive and Negative Affect Schedule (PANAS, Krohne et al., 1996), and the BDI (Hautzinger et al., 2006).

### Procedure

After completion of questionnaires and electrode attachment, pain thresholds were assessed. Then, seven startle probes were presented every 7–14 s to habituate initial startle reactivity (Blumenthal et al., 2005). The experiment consisted of four experimental phases interspersed by online ratings.

During the *exploration phase*, participants actively explored CTX+ and CTX− for 2 min each using a joystick.

During two identical *acquisition phases (ACQ 1 and 2)*, participants were passively guided through the virtual offices on two pre-recorded paths. Paths started from the corridor (inter-trial interval, ITI; lasting about 20 s, entered one virtual room, in which participants remained for 60 s and ended with the exit from the office. Participants entered each room three times in a pseudo-randomized order so that the same room was never entered in more than two consecutive trials. Only CTX+ was paired with one to three US during each trial, resulting in six US per acquisition phase. Altogether, 32 startle probes were delivered (six startle probes per context and four during the ITI per acquisition phase). The shortest time interval between two startle probes, between probe and US or between two US was 10 s (see Grillon et al., 2006), and these stimuli were never delivered during the first and the last 7 s of each room visit to prevent possible associations with the doors.

During the *generalization phase* (GEN), participants were guided through CTX+, CTX− and G-CTX, three times each. The sequence of context presentation was pseudo-randomized. No US were delivered. Six startle probes were presented per room and five during the ITI.

After each phase (Exploration, ACQ1, ACQ2, GEN), ratings of valence, arousal, anxiety, and contingency were assessed (the latter not after Exploration).

### Data Reduction and Statistical Analysis

Physiological responses were recorded continuously with a V-Amp 16 (Version 1.03.0004, BrainProducts Inc., 1,000 Hz sampling rate, 50 Hz notch-filter). Startle responses were registered as electromyographic activity of the left *orbicularis oculi* muscle with 5 mm Ag/Ag-Cl electrodes (see Blumenthal et al., 2005). Ground and reference electrodes were adhered over the right and the left mastoids, respectively. Impedance of the electrodes was kept below 10 kΩ. Startle data was offline filtered using 28 Hz low cut-off and 400 Hz high cut-off filters, then rectified and smoothed (50 ms moving average) and segmented from 50 ms before and 1,000 ms after startle probe onset and finally baseline corrected. Startle amplitude was defined as the maximum of the integrated response curve relative to baseline within 20 ms to 120 ms after probe onset. We scored responses manually and excluded trials with excessive baseline shifts or movement artifacts (signal ≥5 μV). Startle magnitude was averaged for each condition (CTX+, CTX−, G-CTX, and ITI), separately for acquisition and generalization, but at least two valid responses per condition were required for inclusion in further analysis. Participants with a mean startle magnitude <5 μV were excluded as non-responders (*n* = 3). Raw data were within-subjects transformed in z-scores and then in T-scores. Responses to the contexts were calculated as differences in startle magnitude during the visit of the respective context minus preceding ITI responses to control individual baseline differences (Blumenthal et al., 2005).

SCL was recorded with two 8 mm Ag/AgCl electrodes, fixed on the palm of the non-dominant hand. We applied a 1 Hz high cut-off filter offline and then averaged SCL separately for each condition across stay in a virtual context (i.e., 60 s excluding 10 safter US presentations. Finally, SCL data were square root transformed (Boucsein et al., 2012). No participant had to be excluded as non-responders (mean SCL < 0.02 μS).

### Data Analysis

Statistics were performed with SPSS (Version 23.0, SPSS Inc., Chicago, IL, United States). Rating and physiological data were analyzed separately for exploration, ACQ and GEN with repeated-measures analysis of variances (ANOVAs) with between-subjects factor group (PA and HC) and within-subject factor context (exploration and acquisition phase: CTX+, CTX−; GEN: CTX+, CTX+, G-CTX); ANOVAs of acquisition additionally considered the within-subject factor phase (ACQ1, ACQ2). To follow up significant Group × Context interactions, we tested group differences on the basis of* a priori* defined discrimination indices as differences to CTX−; for ACQ, an increased discrimination index of CTX+ minus CTX− indicates better context discrimination and stronger acquisition of conditioned anxiety; for GEN, an increased discrimination index of CTX+ minus CTX− (testCON) indicates more persisting anxiety responses, and an increased discrimination index of G-CTX minus CTX− (testGEN) indicates increased generalization of anxiety.

Significance level was *p* < 0.05, and the Greenhouse-Geisser correction (GG-ε) was applied if necessary. Effect sizes were indicated by partial η^2^.

## Results

### Demographics and Clinical Characteristics

As summarized in Table 1, groups are comparable in gender ratio, age, objective and subjective US intensity, and contingency awareness (all *p*s > 0.191). As expected, PA suffered from more PA (*F*_(1,42)_ = 57.18, *p* < 0.001), more severely panic symptomatology (*F*_(1,42)_ = 66.69, *p* < 0.001) and panic cognitions (*F*_(1,42)_ = 69.95, *p* < 0.001), and avoided significantly more panic-associated situations (*F*_(1,42)_ = 38.23, *p* < 0.001) than HC. Furthermore, PA scored higher in trait anxiety (*F*_(1,42)_ = 10.85, *p* = 0.002), anxiety sensitivity (*F*_(1,42)_ = 17.80, *p* < 0.001), and BDI (*F*_(1,42)_ = 23.80, *p* < 0.001).

**Table 1:** Demographic and psychometric data of participants with panic attacks (PA) and healthy controls (HC).

	PA (*n* = 21)		HC (*n* = 22)		*χ*^2^, *F*	*p*
	*M*	*SD*	*M*	*SD*		
Gender	6 females		10 females		1.31	0.252
Awareness	16 (76%)		20 (90%)		1.71	0.191
Age in years	28.38	10.11	25.68	6.93	1.05	0.311
US intensity (mA)	1.94	0.90	1.99	1.39	0.02	0.879
US intensity rating	5.33	1.46	5.18	1.01	0.16	0.693
STAI Trait	40.90	8.69	33.41	6.06	10.85	**0.002**
ASI	24.71	10.96	12.50	7.83	17.80	**<0.001**
BDI	8.95	4.98	3.05	2.66	23.80	**<0.001**
PFS	3.10	1.92	0.00	0.00	53.17	**<0.001**
PASQ	42.38	24.36	0.00	0.00	66.69	**<0.001**
PACQ	21.95	12.32	0.00	0.00	69.95	**<0.001**
AQ	17.67	13.41	0.00	0.00	38.23	**<0.001**

*ASI, Anxiety Sensitivity Index; STAI, State-Trait-Anxiety-Inventory; BDI, Beck Depression Inventar; PFS, Panic Frequency Scale; PASQ, Panic Attack Symptoms Questionnaire; PACQ, Panic Attack Cognitions Questionnaire; AQ, Avoidance Questionnaire. Bold values indicates significant values*.

### Exploration Phase

**Figure 1:**
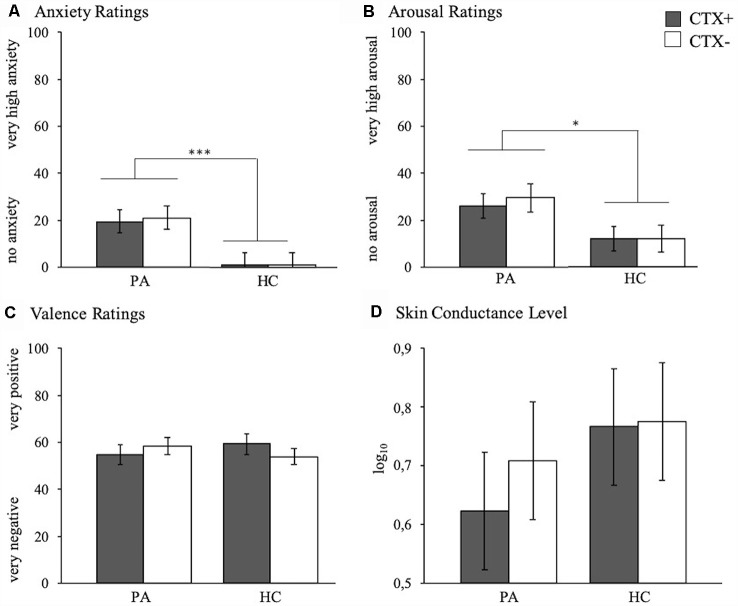
Anxiety **(A)**, arousal **(B)**, and valence **(C)** ratings as well as skin conductance level (SCL) **(D)** of participants with panic attacks (PA, left bars) and healthy controls (HC, right bars) to the CTX+ (gray) and the CTX− (white) during the exploration phase. Depicted are mean responses (means and standard errors). *p < 0.050, ***p < 0.001.

Analyses revealed no significant effects involving the factor context, that is contexts were rated as equivalent in valence (*F*_(1,41)_ = 0.99, *p* = 0.327, $\eta_{\text{p}}^{2}$ = 0.023), arousal (*F*_(1,41)_ = 0.00, *p* = 0.998, $\eta_{\text{p}}^{2}$ = 0.000), and anxiety (*F*_(1,41)_ = 0.621, *p* = 0.435, $\eta_{\text{p}}^{2}$ = 0.015), and they elicited similar SCLs (*F*_(1,41)_ = 3.14, *p* = 0.084, $\eta_{\text{p}}^{2}$ = 0.071). However, PA compared to HC reported overall higher arousal (*F*_(1,41)_ = 5.68, *p* = 0.022, $\eta_{\text{p}}^{2}$ = 0.122) and anxiety (*F*_(1,41)_ = 16.01, *p* < 0.001, $\eta_{\text{p}}^{2}$ = 0.281). Figure 1 depicts these findings.

### Acquisition Phase

First, we found significant context effects indicating successful conditioning for all dependent variables, i.e., valence (*F*_(1,41)_ = 18.85, *p* < 0.001, $\eta_{\text{p}}^{2}$ = 0.315), arousal (*F*_(1,41)_ = 29.93, *p* < 0.001, $\eta_{\text{p}}^{2}$ = 0.422), anxiety (*F*_(1,41)_ = 8.10, *p* = 0.007, $\eta_{\text{p}}^{2}$ = 0.165), contingency rating (*F*_(1,41)_ = 169.27, *p* < 0.001, $\eta_{\text{p}}^{2}$ = 0.805), startle responses (*F*_(1,41)_ = 5.06, *p* = 0.030, $\eta_{\text{p}}^{2}$ = 0.110), and SCL (*F*_(1,41)_ = 6.08, *p* = 0.018, $\eta_{\text{p}}^{2}$ = 0.129).

**Figure 2:**
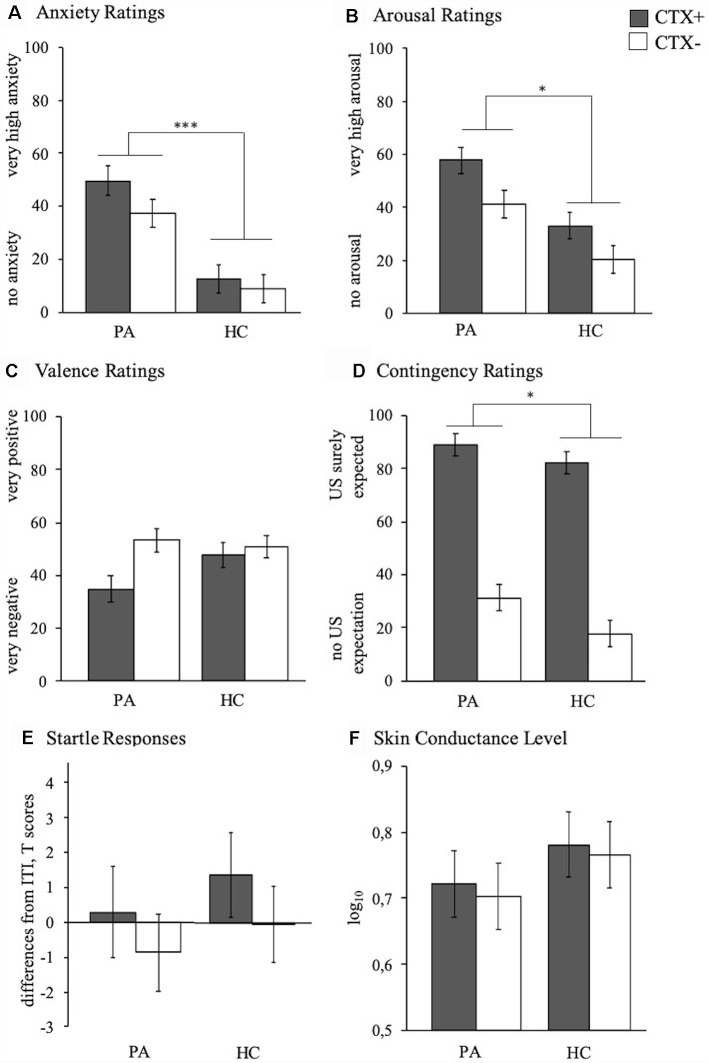
Anxiety **(A)**, arousal **(B)**, valence **(C)**, and contingency **(D)** ratings as well as startle amplitude **(E)** and SCL **(F)** of participants with PA (left bars) and HC (right bars) to the CTX+ (gray) and the CTX− (white) during the acquisition phase. Depicted are mean responses (means and standard errors). *p < 0.050, ***p < 0.001.

Second, as depicted in Figure 2, PA vs. HC exhibited generally increased anxiety (*F*_(1,41)_ = 26.24, *p* < 0.001, $\eta_{\text{p}}^{2}$ = 0.390), arousal (*F*_(1,41)_ = 13.78, *p* = 0.001, $\eta_{\text{p}}^{2}$ = 0.252), and contingency ratings (*F*_(1,41)_ = 8.04, *p* = 0.007, $\eta_{\text{p}}^{2}$ = 0.164), while groups did not differ in valence ratings or both physiological measures (all *p*s > 0.382).

Finally, a significant Context × Group interaction for valence ratings (*F*_(1,41)_ = 10.25, *p* = 0.003, $\eta_{\text{p}}^{2}$ = 0.200) revealed a stronger differential responding of individuals with PA compared to HC (Figure 4C, ACQ).

**Figure 3:**
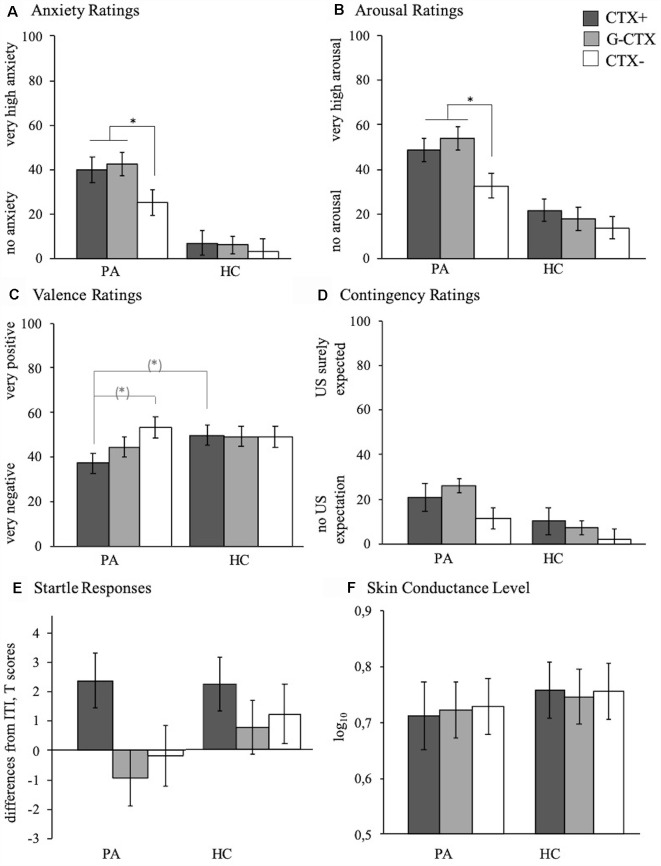
Anxiety **(A)**, arousal **(B)**, valence **(C)**, and contingency **(D)** ratings as well as startle amplitude **(E)** and SCL **(F)** of participants with PA (left bars) and HC (right bars) to the CTX+ (gray) and the CTX− (white) during the generalization phase (GEN). Depicted are mean responses (means and standard errors). *p < 0.050, (*)indicates only marginally significant results.

**Figure 4:**
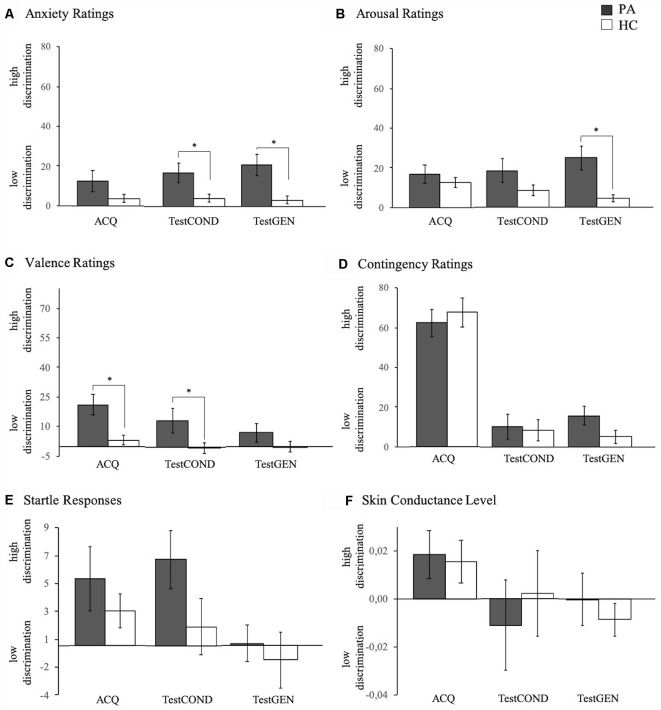
Anxiety **(A)**, arousal **(B)**, valence **(C)**, and contingency **(D)** ratings as well as startle amplitude **(E)** and SCL **(F)** of participants with PA (gray) and HC (white) during the acquisition phase and the generalization test phase. Depicted are mean differential responses (means and standard errors) relative to CTX−, i.e., for acquisition (ACQ) CTX+ minus CTX−, and for generalization CTX+ minus CTX− (testCOND) and G-CTX minus CTX− (testGEN). *p < 0.050.

### Generalization Phase

Analyses revealed for both anxiety and arousal ratings significant context effects (anxiety: *F*_(2,82)_ = 12.93, *p* < 0.001, $\eta_{\text{p}}^{2}$ = 0.240; arousal; *F*_(2,82)_ = 14.85, *p* < 0.001, $\eta_{\text{p}}^{2}$ = 0.266), significant group effects (anxiety: *F*_(1,41)_ = 22.86, *p* < 0.001, $\eta_{\text{p}}^{2}$ = 0.358; arousal: *F*_(1,41)_ = 24.08, *p* < 0.001, $\eta_{\text{p}}^{2}$ = 0.370), and significant Context × Group interactions (anxiety: *F*_(2,82)_ = 6.35, *p* = 0.003, $\eta_{\text{p}}^{2}$ = 0.134; arousal: *F*_(2,82)_ = 5.90, *p* = 0.005, $\eta_{\text{p}}^{2}$ = 0.126), and for valence ratings a marginally significant Context × Group interaction (*F*_(2,82)_ = 2.56, *p* = 0.087, $\eta_{\text{p}}^{2}$ = 0.083; Figure 3).

Further analyses of the interaction effects indicated that PA vs. HC showed overgeneralization of conditioned anxiety as indicated by stronger responses to G-CTX relative to CTX− for anxiety (*t*_(41)_ = 3.05, *p* = 0.004; Figure 4A; testGEN) and arousal ratings (*t*_(41)_ = 3.34, *p* = 0.002; Figure 4B; testGEN).

Following-up the interaction effects regarding maintenance of conditioning effects revealed for PA vs. HC increased responses to CTX+ relative to CTX− for anxiety ratings (*t*_(41)_ = 2.42, *p* = 0.020; Figure 4A; TestCON) and valence ratings (*t*_(41)_ = 2.09, *p* = 0.043; Figure 4C; TestCON) indicating increased maintenance of conditioning effects.

Moreover, we found significant between-group effects for the CTX+ and the CTX−. More precise, PA individuals show higher anxiety and arousal ratings for the anxiety context (anxiety: *t*_(40)_ = 3,771, *p* = 0.001; arousal: *t*_(40)_ = 2,397, *p* = 0.021) and the safety context (anxiety: *t*_(40)_ = 3,490, *p* = 0.001; arousal: *t*_(40)_ = 2,256, *p* = 0.030) as compared to HC.

No interaction effects, but main effects of context were found for contingency ratings (*F*_(2,82)_ = 5.72, *p* = 0.005, $\eta_{\text{p}}^{2}$ = 0.122) and startle responses (*F*_(2,82)_ = 6.91, *p* = 0.002, $\eta_{\text{p}}^{2}$ = 0.144); both groups expected the US to occur in CTX+ (*F*_(1,41)_ = 5.25, *p* = 0.027, $\eta_{\text{p}}^{2}$ = 0.114) and in G-CTX (*F*_(1,41)_ = 13.29, *p* = 0.001, $\eta_{\text{p}}^{2}$ = 0.245) more likely than in CTX− indicating generalization, and both groups exhibited stronger startle responses in CTX+ compared to both CTX− (*F*_(1,41)_ = 7.04, *p* = 0.011, $\eta_{\text{p}}^{2}$ = 0.147) and G-CTX (*F*_(1,41)_ = 12.66, *p* = 0.001, $\eta_{\text{p}}^{2}$ = 0.236) indicating maintenance of conditioned anxiety.

A main effect of group (*F*_(1,41)_ = 5.16, *p* = 0.028, $\eta_{\text{p}}^{2}$ = 0.112) for contingency ratings indicates a generally increased US expectancy in PA compared to HC. Analyses for SCL revealed no significant effects (all *p*s > 0.619).

## Discussion

The current study compared individuals with PA with HC regarding conditioning and generalization of contextual anxiety with the hypotheses that individuals with PA show heightened and persevered context conditioning as well as generalized anxiety responses. First, results revealed successful acquisition of contextual anxiety in both groups indicated by discriminative responses to the anxiety vs. the safety context for anxiety, arousal, valence and contingency ratings and for startle responses and SCLs. These results confirm the VR-paradigm’s success in inducing contextual anxiety (Baas et al., 2004, 2008; Andreatta et al., 2015a, 2017; Genheimer et al., 2017).

Second, PA compared to HC exhibited heightened contextual conditioning, i.e., greater differential responding to CTX+ vs. CTX− for valence ratings after acquisition and for anxiety and valence ratings after the generalization test. As no US was delivered during the GEN, extinction learning may have been initiated during this phase (Milad and Quirk, 2012). Considering the significant main effect of context in ratings that were collected after the GEN, we conclude that this extinction learning was slow and that the implemented trials were not sufficient for extinction. We conclude that individuals with PA, as high trait anxious individuals (Glotzbach-Schoon et al., 2013b), are prone to associate unpredictable threats with a context and to continuously respond with increased anxiety to this context as compared to a safety context, at least on an explicit evaluative level.

Third, we revealed stronger generalization of contextual anxiety in PA than in HC as indexed by anxiety and arousal ratings. This first observation of overgeneralization of contextual anxiety related to PA extends previous reports of overgeneralization of cued fear in anxiety (Lissek and Grillon, 2010; Lissek et al., 2014) and PDs (Lissek et al., 2010). Notably, such over-generalization in PA may be related to the increased anxiety responses to CTX+ vs. CTX−. As contextual conditioning may be based on configural (the context as whole, e.g., office) and/or elemental (the single elements, e.g., chair, desk, etc., Rudy, 2009) representations, it might be speculated that the increased responding to the CTX+ and the G-CTX relative to CTX− have the same cause, increased responding to threat-related elements.

Fourth, participants with PA compared to HC reported overall higher levels of arousal and anxiety as well as generally higher contingency ratings throughout the experiment. These findings validate the groups’ characteristics and confirm the assumption that PD patients are characterized by exaggerated threat appraisal (Beck and Clark, 1997; Beck et al., 2005) and a tendency to overestimate the likelihood of threat (Amrhein et al., 2005; Lissek et al., 2010; Wiemer and Pauli, 2016a,b).

Critically, these findings were not matched by physiological anxiety measures. Specifically, we expected heightened context conditioning reflected in startle responses based on Glotzbach-Schoon et al. (2013c), who examined high vs. low trait-anxious individuals with a similar VR paradigm, and Grillon et al. (2008), who observed increased startle responses in PD patients compared to HC in an unpredictable threat condition. As the observed startle differences descriptively point in the expected direction (see Figure 4E) we expect that future studies with larger samples or diagnosed PD patients will be able to reveal startle effects. Especially since startle probes may impair learning (Sjouwerman et al., 2016), future studies should examine larger samples to have a greater power to reveal physiological effects.

Like in our previous context conditioning and generalization studies in healthy participants, we found context generalization effects for ratings only, but no hint of generalization for startle responses (Andreatta et al., 2015b, 2017). Indeed, this study revealed that startle responses of PA and HC clearly differed between the anxiety and the safety context during acquisition but were very similar for the generalization and the safety context. This may have methodological reasons as our test used one generalization context only which may have been too different from the anxiety context. Therefore, future studies on context conditioning should also use various generalization contexts to allow mapping of generalization gradients as previous cue generalization studies did (e.g., Lissek et al., 2008, 2010, 2014).

In sum, the current study revealed clear context conditioning effects for all participants and generally enhanced anxiety responses in individuals with PA compared to HC as indicated by physiological and verbal measures of anxiety. Importantly, we revealed on an evaluative verbal level heightened acquisition and increased generalization of contextual anxiety in individuals with PA compared to HC and speculate that both effects are risk factors for PD and/or agoraphobia. Such contextual conditioning and generalization processes may be the basis for developing PD and agoraphobia as they may cause anxiety in and avoidance of various contexts (e.g., shopping malls, crowded spaces), and may motivate withdrawal to safety contexts (e.g., the home).

## Ethics Statement

The study was approved approved by the Ethics Committee of the Medical Faculty of the University of Würzburg. All participants gave informed consent. The authors assert that all procedures contributing to this work comply with the ethical standards of the relevant national and institutional committees on human experimentation and with the Helsinki Declaration of 1975, as revised in 2008.

## Author Contributions

DN was responsible for the design of the work and the data collection; carried out the data analysis under the supervision of MA; and drafted the article. MA and PP critically revised the article. PP gave the final approval of the version to be published. All authors contributed to the interpretation of the results.

## Conflict of Interest Statement

PP is shareholder of a commercial company that develops virtual environment research systems. The remaining authors declare that the research was conducted in the absence of any commercial or financial relationships that could be construed as a potential conflict of interest.
